# Fueling Bright Futures: a mixed-methods case study on integrating gardens, canteens, and nutrition training in philippine public schools

**DOI:** 10.1186/s12889-026-28430-w

**Published:** 2026-07-29

**Authors:** Mai A. Hoang, Patricia Isabel A. Matienzo, Diana Jean Umali, Ma. Shiela Anunciado, Lisa Littrell, Susan del Rio, Aman Sen Gupta, Tom van Mourik, Lauren DeVol, Rolf Klemm, Emilita Monville-Oro, Dolly Reario

**Affiliations:** 1 Helen Keller Intl, New York, NY USA; 2 Helen Keller Intl, Manila, Philippines; 3 International Institute of Rural Reconstruction, Cavite, Philippines; 4 Helen Keller Intl, Kathmandu, Nepal; 5 Johns Hopkins Bloomberg School of Public Health, Baltimore, MD USA

**Keywords:** School nutrition, Food environment, School garden, School-based feeding, Scalability, Child dietary behavior, Participatory approaches, Local food systems, Philippines

## Abstract

**Introduction:**

This mixed-methods case study evaluates *Fueling Bright Futures*, an integrated approach involving enhancing school gardens, improving the menu of canteen, and providing nutrition education in three public schools in Cavite, Philippines. The intervention addresses both undernutrition and overweight and obesity, tackling the double burden of malnutrition among Filipino schoolchildren.

**Methods:**

This mixed-methods study was conducted in three public elementary schools in Cavite, Philippines. Baseline and endline assessments (Apr 2024, Feb 2025) included canteen menu audits (per DepEd Order 13), garden inventories, and focus groups with staff and administrators. Effectiveness and scalability were evaluated using the Intervention Scalability Assessment Tool (ISAT) in participatory workshops with policymakers and stakeholders. Qualitative data from all sessions were coded and thematically analyzed via NVivo14 and mapped to ISAT domains.

**Results:**

All the schools made substantial gains in garden output, dietary diversity, and the nutritional quality of canteen offerings. The number of “green” menu items increased sharply (School A: 2—11 per week), whereas the number of “red” foods decreased substantially (School B: 10—2 per week; School C: 13—4). Canteen staff adjusted recipes to use more garden produce, although some packaged snacks persisted. Barriers included limited resources, irregular garden supplies, time constraints, and outside processed food vendors. Strong leadership and community participation elevated acceptability, but infrastructure and sustainability challenges persisted.

**Conclusions:**

Improving and integrating school gardens, improved canteen menus, and staff training can enhance school food environments in resource-limited schools. Success requires strong leadership, partnerships, practical planning, and sustained resources. Ongoing investment and collaboration are critical to building nutrition-friendly school environments that support students’ health and well-being.

**Supplementary Information:**

The online version contains supplementary material available at https://doi.org/10.1186/s12889-026-28430-w.

## Introduction

School-aged children in the Philippines face a dual burden of malnutrition, with persistent undernutrition coexisting with rising rates of overweight and obesity. In 2019, among the 12.7 million children assessed, 13.2% were stunted or wasted. By 2021, obesity affected 10–14% of children aged 5–10 years, up from 9% in 2013 [1]. Stunting remains a major concern, with the Philippines ranking among the highest in the Asia–Pacific region at 27% for children under 5—an indicator that mirrors challenges persisting into school age [1].

A key contributor is poor dietary diversity. Filipino children increasingly consume energy-dense, processed foods, whereas fruit and vegetable intake remains very low (approximately 5–6% of school meals) [1–3]. Moreover, micronutrient deficiencies, socioeconomic disparities, and easy access to unhealthy food inside and outside schools further exacerbate this problem [1].

In this context, the school food environment is both a risk and an opportunity. School-based nutrition initiatives—especially when gardens, healthy canteen offerings, and classroom-based nutrition education—have shown promise in improving children’s diet quality, nutrition knowledge, and even academic outcomes worldwide [4–6]. However, in the Philippines, such interventions are rarely evaluated for scalability and long-term sustainability.

School canteens in the Philippines are central to shaping children’s diets and are regulated through a series of Department of Education (DepEd) policies. These include DepEd Order No. 13, s.2017 on healthy food and beverage choices [7]; DepEd Order No. 8, s.2007 on canteen management [8]; DepEd Order No. 031, s. 2021, on the School-Based Feeding Program [9]; and alongside directives on food safety and nutrition. Collectively, these policies aim to promote healthy eating habits by positioning canteens as accessible platforms for translating national nutrition standards into daily school practices. For many students, particularly in low-resource settings, the quality of canteen offerings represents a substantial portion of their daily nutrition. Studies have shown that school canteens often fail to meet recommended nutrient intakes for children [10], highlighting the critical role of the school environment in influencing dietary behaviors and establishing healthier lifelong habits [9, 10]. 

Despite progress in policy development, the implementation of school nutrition guidelines in the Philippines remains inconsistent due to limited resources, weak monitoring, and competition from external vendors [11]. These challenges highlight the need for integrated, context-specific interventions such as *Fueling Bright Futures*, a pilot intervention that combines improving school gardens, healthier canteen offerings, and hands-on nutrition education.

This paper evaluates the pilot’s implementation and outcomes in public schools with three objectives: (1) assess the effects of garden improvements, canteen reforms, and staff training; (2) explore stakeholder perspectives and operational challenges to sustainability; and (3) examine scalability via the Intervention Scalability Assessment Tool (ISAT) [12]. The use of a mixed-methods case study aims to generate evidence to guide the strengthening and expansion of school nutrition programs in the Philippines and comparable contexts.

## Materials and methods

### Mixed-methods case study design

A mixed-methods design was employed to quantify the effects of the intervention while capturing the complexities of its real-world implementation in schools. Purposive sampling focused on institutions enrolled in the Department of Education’s Integrated School Nutrition Model (ISNM), ensuring that schools had established gardens. This design enabled a comprehensive evaluation of the added value of formally linking school gardens with canteens—a feature not previously emphasized within the ISNM. The quantitative tools used included the ISAT, programmatic checklists, and school food environment surveys, whereas the qualitative methods involved focus group discussions (FGDs) and participatory workshops.

### Setting and participants

The study was conducted in three public elementary schools in Cavite, Philippines: School A (small), School B (medium), and School C (large). These schools were purposively selected to reflect variation in size, resource availability, and community context. All the schools were part of the ISNM and had preexisting gardens and canteens. The study participants included canteen and garden staff, teachers, administrators, and school heads, capturing a comprehensive range of perspectives on the intervention’s implementation.

### Sampling and recruitment methods

A stratified purposive sampling approach was used to ensure representation across small, medium, and large ISNM schools with active gardens and nutrition programs in Cavite Province. Recruitment within each school focused on permanent teachers, canteen staff, garden coordinators, and administrators in SY 2024–2025. Standardized pre- and post-intervention observations fostered consistency and data validity. For broader perspectives, participatory workshops included DepEd officials, school leaders, teachers, and technical experts from UN agencies and civil society, who discussed and scored each ISAT domain to evaluate scalability from multiple sectoral viewpoints.

### Intervention description

The *Fueling Bright Futures* intervention consisted of four integrated components designed to enhance school nutrition holistically:

1. *Garden diversification and adoption of agroecological practices*: This involves diversifying school gardens through the application of agroecological approaches and the cultivation of climate-resilient, indigenous crops while strengthening linkages with school canteens to increase the supply of fresh, locally grown produce and promote long-term sustainability.
2. *Canteen technical assistance*: School canteen menus were reformed through practical, participatory training aligned with DO13, increasing the availability of healthy, policy-compliant meals.
3. *Capacity strengthening and nutrition education for cooks and managers*: Targeted training sessions were provided to school canteen cooks and managers to increase nutrition literacy, increase the quality of meal preparation, and ensure adherence to DO13 and nutrition standards.
4. *Advocacy with school administrators*: Strategic advocacy and engagement encouraged with school principals and administrators to strengthen their commitment to maintaining improved food offerings and provide sustained oversight for DO13 compliance.

All tools were piloted with a small sample of participants to ensure clarity and appropriateness. On the basis of pilot feedback, necessary modifications were made prior to the main study.

### Assessment instruments

- *School Meals and Environment Assessment Tool*: Evaluated compliance with DO13 in three parts: (1) planned menu review against the traffic light system (green daily, yellow 1–2 times/week, red not at all), (2) direct observation of actual offerings, and (3) assessment of the environment for prohibited marketing of red-category foods.
- *School Garden Checklist and Policy Compliance Instruments*: Objectively appraised garden productivity, menu diversity, and adherence to national nutrition guidelines via observations and a checklist.
- *ISAT and ISAT-Aligned Guides*: The ISAT was used to systematically evaluate scalability. To ensure a thorough exploration of all ISAT domains, the research team developed ISAT-aligned guides for FGD and participatory workshops. These guides and the ISAT scoring sheet were developed specifically for this study and have not been published elsewhere. English language versions are provided as follows: the ISAT scoring sheet in Supplementary Material A (Table S1), the FGD guide in Supplementary Material B, and the workshop guide in Supplementary Material C.

### Qualitative data collection procedures

Qualitative insights were collected through the following:

- *Focus Group Discussions*: Three FGDs (one per school), each with 4–6 participants (canteen staff, teachers, administrators), totaling 13 participants. Each FGD lasted approximately 50–60 min.
- *Participatory Workshops and ISAT Assessment*: To assess scalability systematically, two participatory workshops were convened by Helen Keller Intl and the International Institute of Rural Reconstruction (IIRR), which involved approximately 50 stakeholders—including representatives from schools, the DepEd, government agencies (such as the Department of Health, DOST-FNRI), international organizations (UNICEF, World Food Programme), the Philippine Association for Nutrition, and leaders from the three pilot schools. Each three-hour workshop provided broad multidisciplinary input for the use of the ISAT. The participatory workshops were facilitated by project staff with professional backgrounds in public health, dietetics, and agriculture to guide discussions and support an inclusive, informed assessment process.

The ISAT is a structured tool designed to assess a program’s readiness for scale-up, covering ten domains: (1) the problem, (2) the intervention, (3) the strategic political context, (4) evidence of effectiveness, (5) intervention costs and benefits, (6) fidelity and adaptation, (7) reach and acceptability, (8) delivery setting and workforce, (9) implementation infrastructure, and (10) sustainability. During the workshops, the participants collaboratively discussed their implementation experiences within and across these domains.

After group discussion, each organization or sector nominated a representative—typically a senior lead or focal point—to independently score each ISAT domain on a scale of 0–3. Ultimately, 21 nominated representatives submitted individual ISAT ratings. This process ensured that the results reflected balanced, consensus-based, and transparent multisectoral perspectives rather than individual opinions, yielding context-specific assessments of the intervention’s scalability.

All FGDs were audio-recorded and transcribed verbatim in Filipino (Tagalog) and then translated into English. During the participatory workshop, recording was not possible because multiple small group conversations occurred at once; instead, detailed notes were taken in real time. For logistical reasons, the transcripts were not shared with the participants for review. No nonparticipants were present during the workshops or qualitative sessions, and no repeat FGDs were conducted to avoid duplication or respondent fatigue.

Data collection ceased once the intended FGDs were completed, and the research team determined that the information from the participants was sufficiently rich and thematically consistent. Although no additional rounds of data collection were undertaken, the study team judged that thematic saturation had been reached, with no major new themes emerging.

## Data collection and analysis

### Study timing and implementation

The study was conducted over an 11-month period, from April 2024 to February 2025. Data collection procedures were carefully planned to minimize classroom disruption. Each assessment—school canteen menu audits, school food environment observations, and school garden checklists—was scheduled at specific times (e.g., morning classroom sessions, lunchtime, or after lunch) to fit within normal school routines. Strict adherence to privacy and ethical standards, as outlined by DepEd, was maintained throughout the recruitment and data handling process.

### Quantitative analysis

The quantitative data, including garden functionality scores and canteen menu audit results, were summarized with descriptive statistics (means, proportions, temporal trends) and analyzed via statistical software in Excel. For the scalability dimension, the ISAT was administered to a diverse panel of stakeholders representing government, education, and NGO partners. Domain scores were calculated as the means and visually depicted through radar plots to support multidimensional assessment of scalability domains.

### Qualitative analysis

Qualitative data from FGDs and workshops were analyzed thematically using the ISAT framework to ensure that all key scalability domains were addressed. English transcripts and notes were imported into NVivo 14 for systematic coding. Two researchers independently coded the data and discussed the findings. Deductive analysis followed an ISAT-based coding tree (covering fidelity, reach, adaptation, delivery, infrastructure, and sustainability), supplemented by inductive coding for emergent, local insights. Triangulation of qualitative and quantitative data supported a multidimensional understanding of program implementation, barriers, enablers, and scalability prospects.

### Triangulation

We applied an integrated mixed-methods model in which quantitative indicators from garden and canteen audits and ISAT domain scores were systematically juxtaposed with thematically coded qualitative data from FGDs, KIIs, and participatory workshops. This model uses side-by-side comparison of data strands to derive a single, convergent interpretation of effectiveness, implementation barriers and enablers, and scalability across ISAT domains.

## Results

### School and stakeholder profile

The participating schools represented a spectrum of enrollment sizes and baseline nutrition environments. They ranged from rural to peri-urban settings with active local parent groups to larger peri-urban schools with long-standing canteen operations. All three had existing gardens, but their diversity and management systems varied, mirroring the uneven institutional capacity reported in national surveys.

### School gardens

Across all three pilot schools, gardens consistently demonstrated strong performance, with functionality scores ranging from 14 to 16 out of 18 (Table 1). Rather than large shifts, the results highlight sustained management practices, strategic crop diversification, and adaptation to site conditions via climate-smart practices. All gardens cultivated at least three types of vegetables and legumes plus one fruit tree (typically banana or papaya), contributing to both school menu quality and student learning. In addition to overall functionality scores, crop-level changes in fruit and vegetable diversity illustrate how school gardens were adapted to enhance menu planning and year-round food availability; these detailed shifts at each school are presented in Supplementary Material D, Table S2.

Soil health was maintained through compost, manure, and mulching, with School A additionally employing cover crops, especially during the summer season (April—May). Well-fenced beds minimized soil erosion and pest and disease pressure, whereas integration into classroom instruction reinforced gardening as an educational tool. The gardens contained diverse, nutrient-dense crops, used *kakawate* fertilizer trees, and maintained reliable yields with minimal pest or disease issues, making them valuable resources for school food environments. Students, teachers, and parents support daily upkeep and harvesting, complemented by contributions of planting materials and seasonal assistance from parent volunteers, agricultural officers, and other community partners, including local donors and farmers.

**Table 1:** School garden functionality scores (Maximum Score: 18)

School	Baseline (Apr 2024)	Endline (Feb 2025)	Score Range	Classification
School A (small)	14	16	14–16	Moderate-to-high to High
School B (medium)	15	16	15–16	Moderate-to-high to High
School C (large)	14	14	14	Moderate-to-high

*Classification system: 16–18 = high; 13–15 = moderate-to-high; ≤13 = needs improvement

Scores derived from a standardized checklist covering crop diversity, use of organic fertilizers, pest and disease management, garden maintenance, student engagement, and integration into classroom instruction

Canteen managers consistently substituted or enhanced meals with available garden produce, aligning garden planting schedules with menu planning to maximize impact. Garden contributions to canteen dishes often include amaranth, banana, cassava, chili, eggplant, papaya, string beans, and tomato. These crops are most prevalent because of their adaptability to local conditions and nutritional value. For example, micronutrient-dense foods such as papaya, amaranth, chili, and tomato (high in vitamins A, C, iron, and antioxidants), energy-dense foods such as banana and cassava, and protective vegetables such as string bean and eggplant are rich in fiber and some minor vitamins. We also observed some changes in the diversity and types of crops cultivated over time, which were likely affected by variations due to seasons and the availability of planting material.

- *School A (small)*: Maintained broad vegetable selection but reduced fruit diversity from 7 − 5, losing strawberry and dragon fruit but adding chayote, jute mallow, sesame, and rice bean. The continued use of herbs such as oregano has supported pest management.
- *School B (medium)*: Increased fruit diversity by adding mulberry, cotton fruit, and mango at endline, and expanded vegetables to include winged bean and cowpea. New additions replaced less-adapted crops, reflecting a push for year-round food availability.
- *School C (large)*: Lost dragon fruit but gained additional vegetables (pechay, squash, sponge gourd, lima bean), increasing variety, soil fertility, and resilience.

Minimal pest problems were reported, and the active integration of gardening activities into *Edukasyong Pantahanan at Pangkabuhayan*—a compulsory primary school subject in home economics and livelihood skills—helped reinforce practical knowledge and student engagement. Parents and other community members further contributed planting materials, labor, and seasonal support, strengthening both food production and the educational value of the school gardens.

Overall, despite small declines in fruit diversity at some sites, schools demonstrated greater vegetable variety and maintained robust, well-integrated gardens that enhanced canteen menus, supported experiential learning, and deepened community participation.

### School menu and canteen environment shifts

Progress toward compliance with national canteen policy guidelines revealed encouraging improvements alongside persistent challenges, underscoring the complexity of translating nutrition standards into school practice. None of the three schools reached full adherence to DO13 on healthy food and beverage choices, yet quantitative analysis of planned menus revealed marked gains between baseline and endline. School A expanded “green” food offerings weekly from two to eleven items, School B reduced “yellow” items from 30 to nine and “red” items from ten to two, whereas School C lowered “red” items from 13 to four (see Table 2).

**Table 2:** Weekly canteen menu items by DO13 traffic light category pre- and postintervention

School	Time Point	Total Items Per week		
		Green Foods	Yellow Foods	Red Foods
School A	Pre	2	4	2
	Post	11	7	3
School B	Pre	25	30	10
	Post	23	9	2
School C	Pre	12	13	13
	Post	12	12	4

• Green: <3 g saturated fat, < 10 g sugar/carbs, < 120 mg sodium/serving• Yellow: 3–5 g saturated fat, 10–20 g sugar/carbs, 120–200 mg sodium• Red: >5 g saturated fat, > 20 g sugar/carbs, > 200 mg sodium (all 0 g trans fat)

In addition to improving menu frequency according to traffic light category, canteens actively modified the nutritional quality of hot meals. Guided by capacity-building sessions, canteen managers applied menu planning concepts to revise popular dishes with healthier meal preparation methods and ingredients. Substitutions included replacing ultra processed meats with plant-based proteins; enriching meals with eggs and nutrient-dense vegetables from school gardens; and reducing salt, oil, and sugar in dishes such as *pancit*, *champorado*, and *arroz caldo*. These adjustments reflected a practical integration of national nutrition policy with the school garden model and reinforced the use of locally available produce (see Supplementary Material E, Table S3 for detailed recipe modifications).

While these adaptations improved nutrient density in hot meals, a closer analysis revealed significant variation in nutritional quality within each traffic light category. Classification alone often concealed differences between packaged and fresh options. For example, School A’s expansion of “green” items from two to 11 was partially driven by packaged foods (such as corn nuts, banana chips, and cheese wafers), which accounted for 55% of its green offerings at endline. In contrast, Schools B and C demonstrated a shift toward fresh, garden-based offerings: School B reduced packaged green snacks from 20% to 9%, whereas School C modestly lowered them from 62% to 58% (see Table 3).

**Table 3:** Proportion of green foods from packaged snacks pre- and postintervention

School	Pre	Post	Comments
School A	0%	55%	School A increased green hot meal offerings from two to four times weekly but also added multiple green packaged foods.
School B	20%	9%	School B reduced packaged nuts (commercially packaged snacks) from daily to twice weekly and replaced them with fresh produce and boiled eggs.
School C	62%	58%	School C mostly maintained green count; but replaced some packaged snacks with hot meals.

While quantitative analysis revealed improvements in menu composition and reductions in unhealthy items, it also highlighted inconsistencies in how nutrition standards were applied—particularly the reliance on packaged foods to increase “green” offerings. To better understand the drivers of these patterns, qualitative data were used to explore the operational and contextual challenges shaping canteen decision-making. Across schools, canteen managers noted financial constraints, limited preparation time, inadequate facilities, and irregular garden harvests as barriers to full DO13 compliance. Additionally, “vendor leakage”—the sale of DO13 noncompliant foods by external vendors near campuses—further undermined implementation efforts and limited the nutritional impact of school-based reforms.

### Qualitative insights from key stakeholders: ISAT-guided focus group findings

**Fig. 1:**
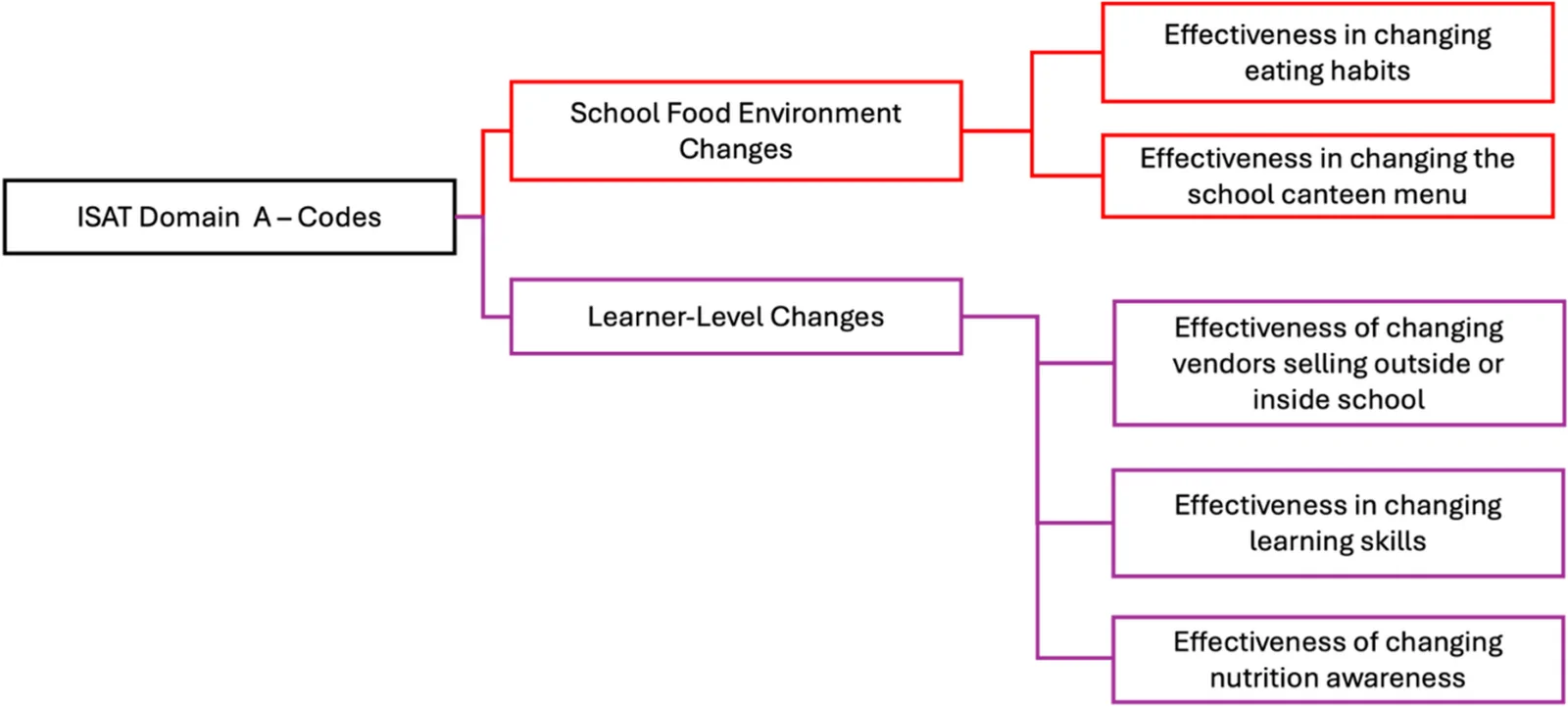
ISAT (Domain A) Illustrative Coding Tree

**Fig. 2:**
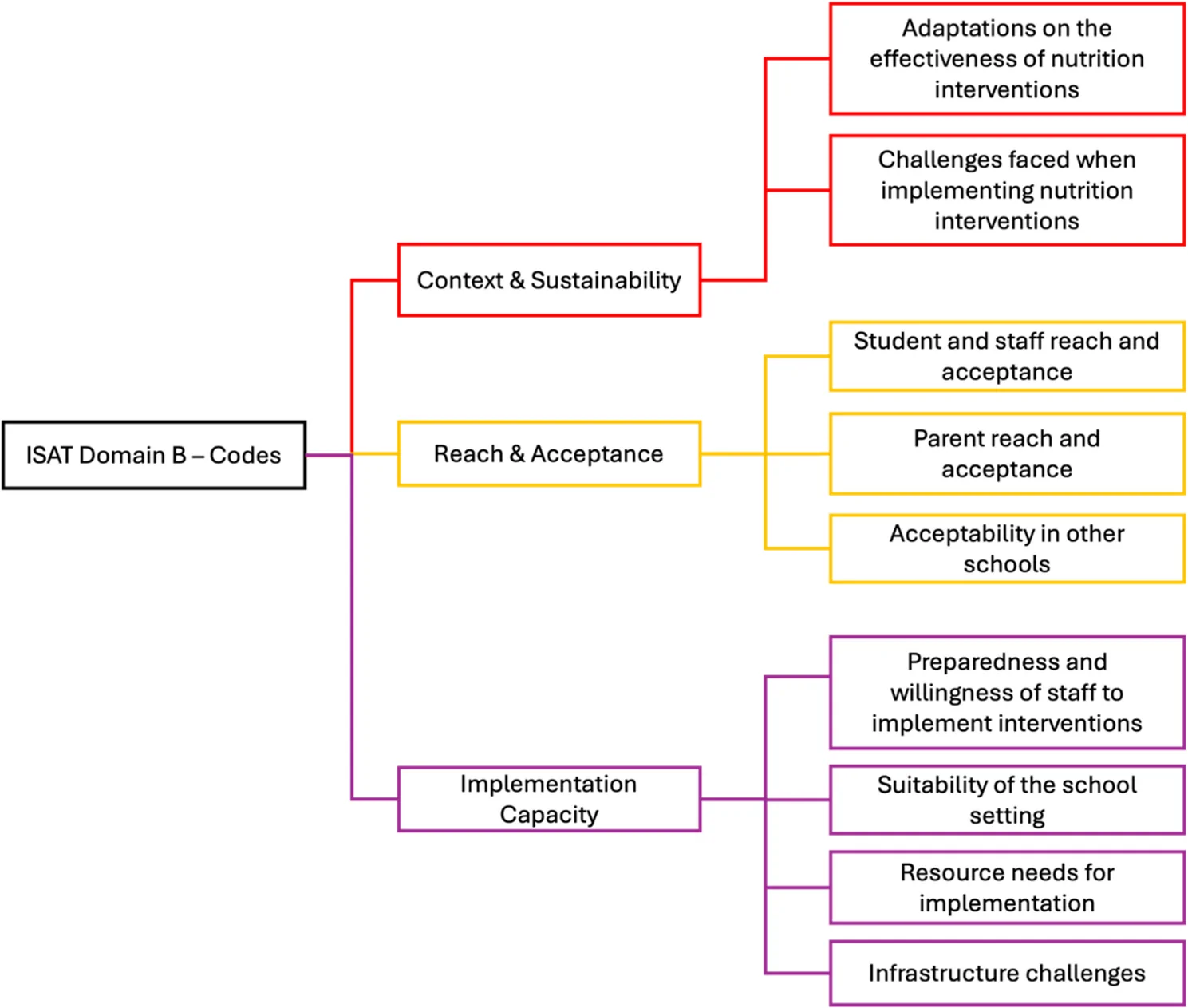
ISAT (Domain B) Illustrative Coding Tree

This section highlights illustrative and key findings from specific ISAT domains; the results for other domains are not included here for brevity and relevance but were covered in the broader analysis. The data were systematically coded by the ISAT domain and subdomain to ensure analytic rigor and transparency (see Figs. 1 and 2 for the coding tree structure). Each theme below corresponds directly to an ISAT domain, illustrating how stakeholder experiences informed the assessment of the intervention’s scalability.

### a. ISAT domain A: evidence of effectiveness

FGDs suggest that *Fueling Bright Futures* contributed to positive shifts in the school food environment and student nutrition behaviors, although the degree and consistency of these outcomes may vary. Several stakeholders observed that replacing processed foods with locally grown fruits and vegetables in canteens resulted in diverse available choices and made healthy food more appealing, especially where menu innovations were implemented. Notably, changes in food perceptions among students took time, with some overcoming initial skepticism.

- *“Initially*,* they thought vegetables were just weeds. Now they’ve discovered that these can be cooked and eaten…They didn’t realize they were edible.”—Canteen staff*,* FGD*,* School C*.
- *“The most impressive activity I’ve seen is what they offer in the canteen. They sell food…using bananas and vegetables…that could be replicated in other schools.” —Canteen staff*,* FGD*,* School C*.

While these improvements were encouraging, the impact extended beyond just food offerings. The participants noted a shift in purchasing habits toward the school canteen and away from outside vendors, as well as the development of students’ practical skills through participation in gardening and food preparation. The extent of these changes depended on student engagement and exposure to new recipes, which seemed to increase curiosity and openness over time.

- *“When they saw [vegetable juice] being prepared*,* they became curious…They weren’t interested before but gradually tried it…vegetables can be used in juice.”—Canteen staff*,* FGD*,* School B*.
- *“Before*,* there were many [vendors]*,* but now there’s only one…[kids] just buy here inside*,* which is fine.” —Canteen staff*,* FGD*,* School B*.

Across ISAT Domain A regarding evidence of effectiveness, these qualitative insights indicate that *Fueling Bright Futures’* interventions have improved both the availability and appeal of healthier foods, while gradually shifting students’ purchasing patterns toward school canteens and away from external vendors. These perceived changes in behavior align with the quantitative gains in sales of green menu items and stable, high garden functionality scores, suggesting that improved supply was accompanied by growing demand. These findings support the interpretation that the piloted interventions can meaningfully strengthen the school food environment, although the magnitude and consistency of behavior change may vary by school.

### b. ISAT domain B: fidelity, adaptation, reach, workforce, and implementation

Domain B of the ISAT framework explores how nutrition interventions such as *Fueling Bright Futures* maintain integrity and adaptability in diverse school settings, how they gain acceptance, and what operational factors and resource gaps affect their scalability.

#### Fidelity and adaptation

Adaptability was essential; success depended on school leadership, garden productivity, and sustained volunteer involvement. Canteens thrived with varied menus and supportive parents.

- *“Income doubled…because the cooked meals are now more delicious.” —Canteen staff*,* FGD*,* School A*.
- *“It still depends on the principal…stakeholders and community members are involved in gardening…”—Canteen staff*,* FGD*,* School A*.

#### Reach and acceptability

Resistance to new foods and pricing decreased over time through adaptation, communication, and the incorporation of vegetables into familiar dishes.

- *“At first…complaints…food was too expensive…students wouldn’t buy it. But eventually*,* they understood…we started blending [vegetables]…they got used to it.” —Canteen staff*,* FGD*,* School B*.
- *“Acceptance increased as students’ preferences evolved.” —Canteen staff*,* FGD*,* School A*.

#### Delivery settings and workforce

Staff and teacher training concerning food safety, labeling, and menu planning was valuable, although effective application depended on practical resource support.

- *“Before*,* I didn’t know how to write and calculate for the green labeling…learned about labels…food safety…menu plan.” —Teacher*,* FGD*,* School C*.

#### Implementation infrastructure

Shortages of basic tools, equipment, and operating funds frequently limited improvements and undermined what staff learned in training.

- *“Cooking equipment like pots*,* pans*,* and knives. We truly need more cooking tools.” —Canteen manager*,* FGD*,* School C*.
- *“The cost of goods has increased…and our expenses have also gone up…”—Canteen manager*,* FGD*,* School C*.

#### Sustainability

Long-term progress was threatened by budget uncertainty and leadership turnover, despite optimism for school gardens and policy support.

- *“That’s what’s not sustainable? …if the canteen management changes…you won’t have the same level of support.” —Project staff*,* FGD*,* School C*.
- *“If the principal is good at doing PR*,* it is easier to obtain support from the parents. When the principal is just always inside their office*,* we had difficulty…”—Canteen manager*,* FGD*,* School B*.

Across ISAT Domain B, FGD participants emphasized that successful implementation relied on strong school leadership, dedicated canteen staff, and active engagement from parents and the community, while equipment gaps and rising food costs limited progress. Adaptations such as blending vegetables into familiar dishes and adjusting prices improved reach and acceptability, highlighting the need for context-specific flexibility. These insights align with ISAT scores for fidelity, reach, workforce, infrastructure, and sustainability, and clarify key operational enablers and barriers to scalability.

### ISAT roundtable participatory assessment

As detailed in the Methods, stakeholders from multiple sectors completed the ISAT to evaluate the scalability of *Fueling Bright Futures*. The following summarizes domain-level findings:

#### Fidelity and adaptation (2.3/3)

Stakeholders highlighted the value of context-specific adaptation and volunteer involvement but emphasized the need for continuous oversight and quality monitoring.

- “Spreading out to different schools…bound to the integration of nutrition education into behavior.”—*DoH Rep*.
- “Programs should intensify monitoring, along with improving WASH…to ensure food safety and maintain hygiene.”—*DepEd central official*.

#### Reach and acceptability (2.3/3)

Initial concerns regarding costs and stakeholder buy-in decreased as parental and student engagement increased; principal leadership and clear orientation were identified as critical for reach and uptake.

- “Children should know the value of healthy food—they need regular exposure to indigenous foods.”—*DepEd division official*.
- “Menu improvements can drive further implementation.”—*DepEd central official*.

#### Delivery setting and workforce (2.3/3)

The commitment of the pilot schools was evident, yet the stakeholders noted that scaling efforts necessitate additional personnel and operational resources.

- “DepEd tried to hire additional canteen personnel to lessen the burden on teachers…”—*DOST-FNRI representative*.
- “If canteen management is removed from teachers…the response may be to outsource to concessionaires…”—*School head*.

#### Implementation Infrastructure (1.7/3)

Infrastructure shortcomings—tools, kitchen equipment, and stable funding—were seen as critical challenges and limits to scale.

- “School gardens and canteens are not that well-funded…virtual sessions…not as effective compared to face-to-face.”—*DepEd regional official*.

#### Sustainability (1.7/3)

While high-level policy support exists, chronic challenges with resourcing and management are seen as barriers to long-term sustainability, necessitating stronger partnerships and ongoing community participation.

- “Improvements…were demonstrated, but scaling demands increased monitoring…as well as strengthened school feeding and canteen programs.”—*DepEd central leader*.
- “Small steps through interventions such as school gardens and dietary guidelines can be impactful.”—*DOST-FNRI representative*.

#### Overall assessment and recommendation

**Fig. 3:**
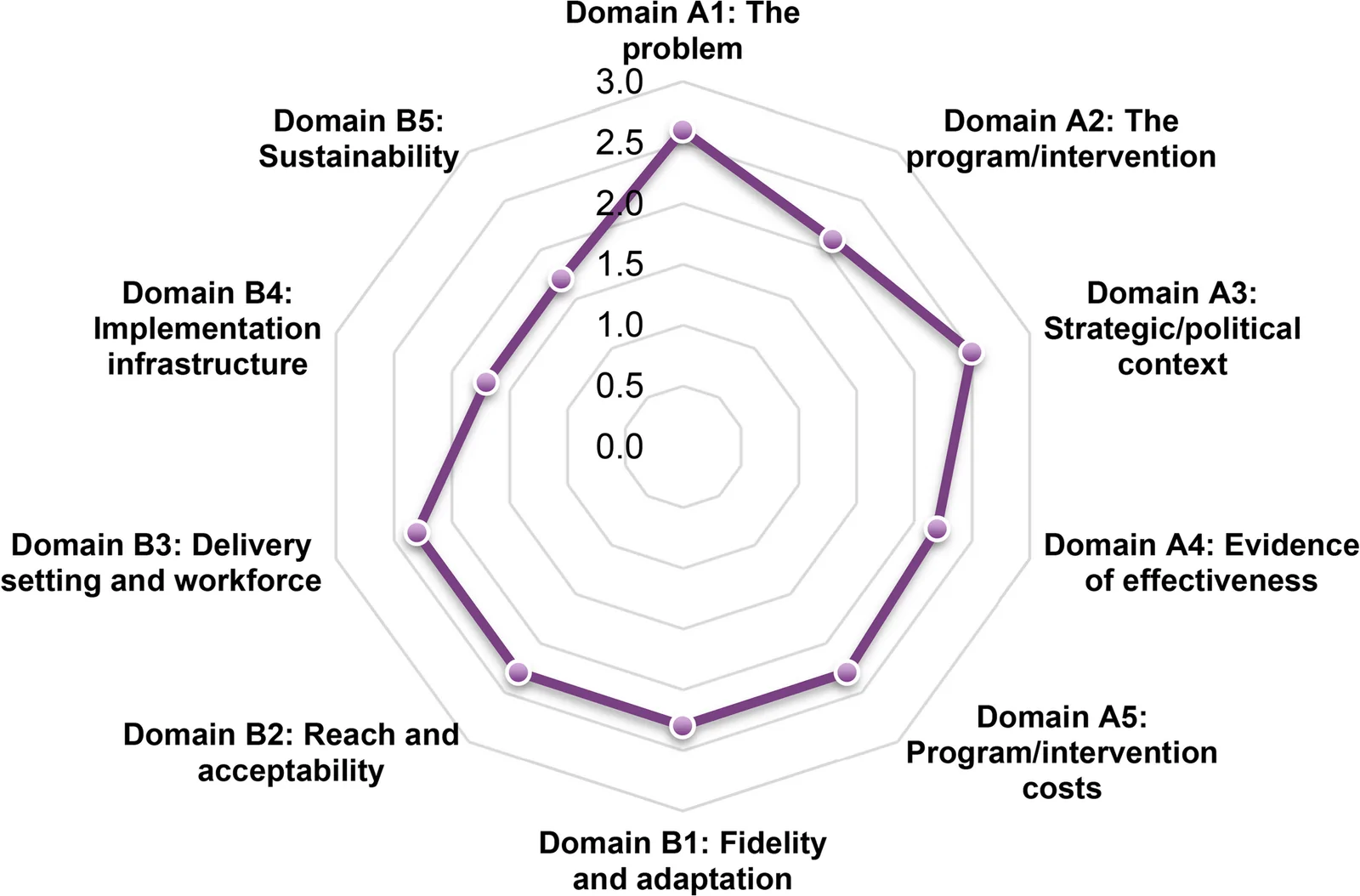
ISAT Domain Ratings for the *Fueling Bright Futures* Pilot

On the basis of ISAT scoring, *Fueling Bright Futures* was considered promising and merits further scale-up, but the evaluation flagged significant opportunities for strengthening the infrastructure and sustainability domains. Stakeholders recommended a phased expansion to additional schools, with best practices, and identified barriers addressed iteratively. See Fig. 3 for ISAT domain ratings and overall pilot assessment.

To make the mixed-methods design and the triangulation between data strands more explicit, Table 4 summarizes how key quantitative indicators are triangulated with corresponding qualitative insights across ISAT domains. This presentation shows changes in school gardens, canteen menus, perceived implementation conditions, and shared judgments on effectiveness, barriers, enablers, and scalability prospects.

**Table 4:** Integrated summary of quantitative and qualitative findings across ISAT domains in the *Fueling Bright Futures* pilot

ISAT domain / objective focus	Key quantitative indicators	Summarized Qualitative insights (FGDs and workshops)	Integrated interpretation
Domain A: Evidence of effectiveness Objective 1: effects on gardens, canteens, staff practices	• Garden functionality scores remained moderate-to-high to high in all schools (14–16/18). • Relative share of green canteen items increased (e.g., School A 2→11/week; School B and C large reductions in red items).	• Staff perceived greater availability and variety of healthy options and reported that healthy foods became more appealing to students over time. • Increased sourcing of healthy fresh foods from school gardens, may reduce food purchase costs and increase sustainability. • Some schools noted shifts in purchasing from outside vendors to school canteens.	Quantitative improvements in menu quality and garden performance are reinforced by reported changes in student perceptions and purchasing patterns, indicating a stronger and more health-promoting school food environment. School A’s large increase in green items was driven mainly by packaged snacks, reflecting infrastructure, time, and garden-supply constraints that favored convenient but compliant options. In contrast, Schools B and C shifted more toward fresh, garden-based items, consistent with qualitative reports of more diverse gardens, leadership, and active community engagement.
Domain B: Fidelity, adaptation, and reach Objective 2: implementation barriers/enablers	• ISAT fidelity and adaptation score ≈ 2.3/3; reach and acceptability ≈ 2.3/3.	• Success linked to strong principals, active volunteers, and the ability to adapt menus (e.g., blending vegetables into familiar dishes, adjusting prices).- Initial resistance to new foods and pricing decreased as familiarity grew among students and parents.	Moderate-to-high ISAT scores are consistent with reports that the model is acceptable and adaptable when leadership and community support are present, highlighting adaptation and communication as key enablers of reach.
Domain B: Delivery setting, workforce, and infrastructure Objective 2: implementation barriers/enablers	• ISAT delivery setting/workforce ≈ 2.3/3; implementation infrastructure ≈ 1.7/3.	• Training improved menu planning, labelling, and food safety knowledge among teachers and canteen staff. - Persistent shortages of kitchen tools and operating funds limited full application of training.	Ratings and narratives converge to show that workforce capacity has improved but is constrained by weak infrastructure, implying that further scale-up will require investments in equipment and operating budgets.
Domain B: Sustainability and scalability Objective 3: Scalability	• ISAT sustainability score ≈ 1.7/3; overall assessment that the intervention is “promising and merits further scale-up.”	• Stakeholders and roundtable participants expressed optimism about gardens and policy support but raised concerns about leadership turnover, budget uncertainty, and competing vendor pressures.	Lower sustainability scores, together with concerns about leadership and financing, indicate that while the model is promising for phased expansion, long-term impact will depend on institutionalizing resources and governance arrangements for dedicated staff time.

## Discussion

Global evidence consistently demonstrates that integrated school nutrition programs—including school feeding, local food procurement, nutrition education, and supportive policy frameworks—can improve child health and educational outcomes [13–15]. However, the effectiveness of these programs in reducing chronic undernutrition at various scales, such as underweight, remains unclear. Recent large-scale studies emphasize the need for more rigorous research to clarify their impact on a broader range of health and academic outcomes [16].

Experiences from Ghana and Bolivia highlight the value of connecting school meals to local food systems, engaging community stakeholders, and integrating nutrition education into curricula [17, 18]. Initiatives such as local food procurement, school gardens, and collaboration across sectors have been linked to improved program sustainability and alignment with national objectives [15]. The World Food Programme’s latest review revealed that setting clear nutrition goals, sourcing foods locally, and fostering intersectoral partnerships strengthen program outcomes, although persistent barriers remain. These include fragmented government responsibilities and limited capacity for monitoring nutritional impacts [15].

The findings from the *Fueling Bright Futures* pilot echo international trends: improving and integrating school gardens, food environment improvements, engagement of key leadership and staff, and participatory nutrition education enhanced menu quality and student dietary diversity. Fresh and diverse foods from school gardens, mandated by policies such as Gulayan sa Paaralan (GPP) [19] and DO 8, were directly incorporated into meals. The active participation of canteen staff and volunteers not only improved the quality of food offered but also enabled cost savings, thereby promoting program sustainability.

At the same time, the results suggest that implementation varied meaningfully across the three schools and was strongly shaped by local context. Although School A recorded the largest increase in green menu items, over half of these were packaged snacks (e.g., corn peanuts, banana chips, and cheese wafers) rather than fresh or garden-based foods. This pattern reflects implementation constraints identified through ISAT scoring and qualitative data, including limited kitchen infrastructure, time constraints for food preparation, and irregular garden supply. As such, the reliance on packaged “green” items represents policy-compliant adaptation under constrained conditions, rather than a contradiction of effectiveness findings.

In contrast, Schools B and C showed a stronger shift toward fresh, garden-based green items and larger reductions in red foods, consistent with quantitative and qualitative accounts of more diverse gardens, stronger principal leadership, motivated canteen staff, and active parent or community engagement. These contextual factors influenced, summarized in the integrated quantitative–qualitative table (Table 4), indicate that school-level differences in leadership, garden diversity, infrastructure, and stakeholder involvement influenced whether schools were able to translate policy compliance into increased availability of fresh, minimally processed foods, or relied more heavily on packaged but compliant alternatives. These findings suggest that School B and C operated within a more implementation-ready context, enabling deeper integration of garden produce into daily menus.

The ISAT findings further contextualize these differences. Moderate-to-high ratings for fidelity, adaptation, reach, and workforce (≈ 2.3/3) suggest that stakeholders viewed the model as generally acceptable and adaptable, particularly where school leadership was strong, and volunteers could be mobilized. However, lower scores for implementation infrastructure and sustainability (≈ 1.7/3) underscore persistent gaps in basic equipment, operating funds, and long-term resourcing, which participants described as limiting the depth and durability of changes, especially in schools with fewer assets or weaker management systems. Together, these findings suggest that scaling Fueling Bright Futures will necessitate contextualized and tailored strategies that account for infrastructure investments and school leadership engagement.

### Limitations and generalizability

Despite valuable insights, the findings are limited by the focus on only three schools within one province and a short intervention period, affecting generalizability and the assessment of long-term impact. Potential bias from self-reported data and variations in resources or engagement could also influence the results. Future studies with larger, more diverse samples and longer follow-up periods are needed to better assess the scalability and durability of these approaches.

## Conclusions

The *Fueling Bright Futures* pilot in Cavite offers valuable, context-specific evidence that school nutrition programs can serve as effective public health interventions in the Philippines and similar global settings —in resource-limited settings. By integrating climate-resilient school gardens, practical canteen policy guidance, and participatory nutrition education and skills training for staff, the pilot led to clear improvements in school food environments and dietary diversity in canteen offerings.

However, the *Fueling Bright Futures* experience reiterates a lesson found internationally: Successful implementation depends on strong leadership, adequate infrastructure, and sustained community engagement, while scalability will require continued investment, institutional support, and shared accountability among food system actors. Participatory assessments using the ISAT confirmed persistent challenges—such as gaps in infrastructure and staff capacity—but also highlighted the importance of shared accountability among stakeholders in overcoming these barriers and promoting sustainability.

Looking forward, a strengthened school food environment in the Philippines will require greater emphasis on making tools such as the DO13 traffic light system practical and accessible for everyday use. Specifically, canteen managers and school staff would benefit from hands-on job aids and simple, easy-to-understand tools that guide menu planning and food categorization, ensuring that the policy can be realistically and consistently applied in diverse school settings. To address nutritional gaps and support efficient planning, open-access tools such as the Partnership for Child Development School Meals Planner and the PLUS School Menus have been developed to help program planners design meals that are nutritionally balanced and tailored to reference nutrient intakes, especially in resource-poor settings.

Institutionalizing nutrition education as a regular, classroom-based activity is essential, as research shows that building food literacy promotes healthy eating habits over the long term [6]. Fostering partnerships among community and school stakeholders will help sustain and scale these gains. Ultimately, the Cavite pilot underscores a broader lesson: system-level, policy-aligned, and multisectoral approaches—grounded in supportive school environments and classroom learning—are essential to create resilient, equitable food and nutrition systems that advance child health and educational outcomes [16, 20].

## Supplementary Information

Supplementary Material 1.

Supplementary Material 2.

Supplementary Material 3.

Supplementary Material 4.

Supplementary Material 5.

## Data Availability

Data and materials supporting this study are available upon reasonable request from the Principal Investigator. Access will be granted following completion of a data sharing agreement with Helen Keller Intl.
