# Supplementary material for: Fueling Bright Futures: a mixed-methods case study on integrating gardens, canteens, and nutrition training in philippine public schools
Source: BMC Public Health. 2026 Jul 29;26:3063. doi: 10.1186/s12889-026-28430-w (PMC13700392; doi:10.1186/s12889-026-28430-w)
Supplement: Supplementary file 1 [file 12889_2026_28430_MOESM1_ESM.docx]

# Supplementary Material A: ISAT Scoring Sheet

**Table S1: Intervention Scalability Assessment (ISAT) Tool**

| **Domain/Question** | | **N/A** | **Not at all (0)** | **To a small extent (1)** | **Somewhat (2)** | **To a large extent (3)** |
| --- | --- | --- | --- | --- | --- | --- |
| **Domain A1: The Problem** | | | | | | |
| 1 | Is the problem of sufficient concern to warrant scale-up of an (the) intervention/program to address it? |  |  |  |  |  |
|  | **Total score Domain A1** |  |  |  |  |  |
| **Domain A2: The Program/intervention** | | | | | | |
| 2 | Will the outcomes delivered by this program/intervention address the needs of the target group (and/or) problem? |  |  |  |  |  |
|  | **Total score Domain A2** |  |  |  |  |  |
| **Domain A3: Strategic/political context** | | | | | | |
| 3 | Is addressing the problem consistent with policy/strategic directions or priorities? |  |  |  |  |  |
| 4 | Will scaling up this program/intervention up be strategically useful to funders/ funding agency? |  |  |  |  |  |
|  | **Total score Domain A3** |  |  |  |  |  |
| **Domain A4: Evidence of effectiveness** | | | | | | |
| 5 | Is there compelling evidence from the literature to indicate that the program/intervention is effective in addressing the problem in the target population? |  |  |  |  |  |
|  | **Total score Domain A4** |  |  |  |  |  |
| **Domain A5: Intervention costs** | | | | | | |
| 6 | Is there evidence that the benefits of the intervention exceeded the costs? |  |  |  |  |  |
|  | **Total score Domain A5** |  |  |  |  |  |
| **Domain B1: Fidelity and adaptation** | | | | | | |
| 7 | Will the core components of the school nutrition interventions (canteen manager training, interactive learning activities, and school gardens) maintain their effectiveness when implemented in different Filipino school contexts? |  |  |  |  |  |
| 8 | If adaptations to the nutrition interventions are needed for different school contexts (urban vs. rural, large vs. small schools), will these modifications likely maintain positive impacts on student nutrition? |  |  |  |  |  |
| 9 | Can the quality and consistency of the nutrition interventions be effectively monitored and maintained when implemented across multiple schools in different regions? |  |  |  |  |  |
|  | **Total score Domain B1** |  |  |  |  |  |
| **Domain B2: Reach and acceptability** | | | | | | |
| 10 | Do the school canteen and school garden interventions have the potential to reach all intended students across different school settings throughout the Philippines? |  |  |  |  |  |
| 11 | Are these school nutrition interventions likely to be acceptable to Filipino students, parents, and school staff across different cultural and socioeconomic contexts? |  |  |  |  |  |
|  | **Total score Domain B2** |  |  |  |  |  |
| **Domain B3: Delivery setting and workforce** | | | | | | |
| 12 | Are Filipino public schools appropriate settings for delivering these nutritional interventions at scale, considering variations in facilities (e.g., kitchens, bandwidth of staff) and resources? |  |  |  |  |  |
| 13 | Are principals, teachers, canteen managers, and cooks appropriate workforce for delivering these nutrition interventions across diverse school settings? |  |  |  |  |  |
| 14 | Will these nutrition interventions be acceptable to school staff in different contexts, considering their existing workloads and responsibilities? |  |  |  |  |  |
| 15 | How well would these nutrition interventions integrate with existing DepEd programs, policies, and school structures across different regions? |  |  |  |  |  |
|  | **Total score Domain B3** |  |  |  |  |  |
| **Domain B4: Implementation infrastructure** | | | | | | |
| 16 | Are the resources, facilities, and systems required for these school nutrition interventions (canteens, gardens, training) feasible across diverse Filipino school contexts? |  |  |  |  |  |
|  | **Total score Domain B4** |  |  |  |  |  |
| **Domain B5: Sustainability** | | | | | | |
| 17 | Can these nutrition interventions be sustainably integrated into regular school operations without creating unsustainable burdens on school administration? |  |  |  |  |  |
| 18 | Is the level of funding, materials, and technical support required for these nutrition interventions sustainable within DepEd's existing budget and resource allocation systems? |  |  |  |  |  |
| 19 | Can principals, teachers, and canteen staff sustain these interventions over time without experiencing burnout or requiring constant external support? |  |  |  |  |  |
|  | **Total score Domain B5** |  |  |  |  | . |
