# Supplementary material for: Fueling Bright Futures: a mixed-methods case study on integrating gardens, canteens, and nutrition training in philippine public schools
Source: BMC Public Health. 2026 Jul 29;26:3063. doi: 10.1186/s12889-026-28430-w (PMC13700392; doi:10.1186/s12889-026-28430-w)
Supplement: Supplementary file 2 [file 12889_2026_28430_MOESM2_ESM.docx]

# Supplementary Materials B

## Endline Focus Group Discussion Guide: School Canteen Cooks, Managers, Teachers

The participants per school are the school principal, school canteen manager, school canteen cook(s), and schoolteachers.

**Maximum participants per school:** 6-8 participants per school

**Objective**

The focus group discussion (FGD) aims to evaluate the impact of recent interventions, such as school trainings related to nutrition, on the practices and perceptions of principals, teachers, canteen managers, and cooks. The discussion will focus on significant changes observed, sustainability of improvements, future aspirations, required resources, and suggestions for enhancing the healthy food environment in schools.

**Duration**

1.5 hour

**Discussion Guide**

1. **Introduction (5 minutes)**

- Welcome participants and introduce the purpose of the FGD.
- Do a friendly warm-up and ice-breaker activity
- Briefly explain the importance of evaluating interventions to improve school nutrition.
- Read the consent form and make sure all participants understand participation is voluntary. Obtain written informed consent.
- Assure participants all information is anonymous.

### Domain A1: The problem

1. What are your thoughts on the malnutrition problem in your school?
2. What specific nutritional challenges do you observe among students?

### Domain A2: The Program/Intervention

3. What key learnings did you gain from the recent project interventions (school canteen manager training, interactive learning activities, and advocacy meetings) and other discussion sessions?

Probe: How have these learnings influenced your daily practices?

4. Can you share specific examples of how the school canteen training has impacted your approach to nutrition in your school?
5. Can you share specific examples of how school gardens have been used in your canteen since the start of this project?

Probe: How do you think the school canteen training will help in shaping children’s eating habits?

### Domain A3: Strategic/Political Context

6. Do you think that the DepEd office will adapt this intervention to scale up to other schools? Why or Why not?

### Domain A4: Evidence of Effectiveness

7. What are the most significant changes or improvements resulting from these interventions or this project?

- Why do you view these changes as significant?

8. Do you think these changes have affected students’ eating habits or overall health?
9. Do you think interactive learning activities to improve nutrition knowledge and decision-making will enhance students' eating habits?

**Domain B1: Fidelity and Adaptation**

10. Do you think any of these interventions can be scaled up to other schools?

Probe: Which specific interventions are scalable and why?

11. If we were to expand this project to other schools, what do you think we should do differently? Why?

### Domain B2: Reach and Acceptability

12. To what extent are the school canteen and school garden interventions reaching all intended students?
13. How well are these interventions being accepted by students, parents, and staff?
14. Do you think this intervention will be acceptable and applicable to other schools? Why or why not?

### Domain B3: Delivery Setting and Workforce

15. How suitable are schools for delivering these nutritional interventions?
16. How prepared and willing are the principal, teachers, school canteen cooks, and managers to implement these interventions?

### D**omain B4: Implementation Infrastructure**

17. What specific resources or infrastructure are needed to implement these interventions effectively?
18. Are there any challenges in maintaining the necessary infrastructure for these interventions?

### Domain B5: Sustainability of Improvements

19. Which improvements do you believe are sustainable in the long term?

Probe: What factors contribute to this sustainability?

20. Are there any practices or changes that you think may not be sustainable? Why?
21. What resources or support do you need to sustain a healthy food environment in your canteen?

Probe: Consider aspects such as funding, training, supplies, manpower or community partnerships.

22. How can the school administration or DepEd assist in these areas?
23. How can the school administration or DepEd assist in these areas?

**Conclusion (5 minutes)**

- Summarize key points discussed during the FGD.
- Thank participants for their valuable insights and contributions.
- Discuss next steps regarding how this feedback will be utilized.
