# Supplementary material for: Fueling Bright Futures: a mixed-methods case study on integrating gardens, canteens, and nutrition training in philippine public schools
Source: BMC Public Health. 2026 Jul 29;26:3063. doi: 10.1186/s12889-026-28430-w (PMC13700392; doi:10.1186/s12889-026-28430-w)
Supplement: Supplementary file 3 [file 12889_2026_28430_MOESM3_ESM.docx]

# Supplementary Materials C

## Reflection Workshop: Agenda and Discussion Guide for Meeting with DepEd Members and ISNM Technical Working Group

**Objective**

The meeting aims to review and evaluate the Fueling Bright Futures interventions under the Integrated School Nutrition Model (ISNM). Participants will discuss key lessons learned, challenges faced, potential solutions, necessary measures for efficacy and sustainability at the school level, and recommendations for future initiatives.

**Duration**

2 hours

**Meeting Agenda**

**1. Welcome and Introductions (10 minutes)**

- Opening remarks by the facilitator.
- Brief introductions of all participants.

**2. Overview of Fueling Bright Futures Interventions (15 minutes)**

- Presentation of the objectives and scope of the Fueling Bright Futures interventions.
- Summary of the ISNM framework and its components.

**3. Key Lessons Learned (20 minutes)**

- Discussion on significant lessons learned from the interventions.
  - What strategies were most effective?
  - How have these lessons influenced current practices within DepEd and ISNM?

**4. Challenges Encountered (20 minutes)**

- Identification of challenges faced during the implementation of the interventions.
  - Resource allocation, stakeholder engagement, or community involvement issues.
- Discussion on how these challenges impacted the effectiveness of the interventions.

**5. Potential Solutions (20 minutes)**

- Brainstorming session on solutions to address identified challenges.
  - Sharing best practices among schools.
  - Strengthening collaboration between DepEd, local government units (LGUs), and other stakeholders.

**6. Efficacy and Sustainability at School Level (20 minutes)**

- Discussion on key elements necessary for ensuring efficacy and sustainability of nutrition interventions at the school level.
  - Training for staff, community engagement, ongoing monitoring.
- Identifying support mechanisms needed for schools to maintain these initiatives over time.

**7. Recommendations for Future Initiatives (20 minutes)**

- Gathering recommendations from participants for enhancing future nutrition interventions.
  - Areas needing additional focus or resources.
- Discussing ways DepEd can enhance its role in supporting schools to promote a sustainable healthy food environment.

**8. Wrap-Up and Next Steps (15 minutes)**

- Summarization of key points discussed during the meeting.
- Outline next steps based on feedback gathered.
- Closing remarks by the facilitator.
