# Supplementary material for: Fueling Bright Futures: a mixed-methods case study on integrating gardens, canteens, and nutrition training in philippine public schools
Source: BMC Public Health. 2026 Jul 29;26:3063. doi: 10.1186/s12889-026-28430-w (PMC13700392; doi:10.1186/s12889-026-28430-w)
Supplement: Supplementary file 4 [file 12889_2026_28430_MOESM4_ESM.docx]

# Supplementary Materials D

**Table S2. Changes in Crop Diversity in Pilot School Gardens from Baseline (April 2024) to Endline (February 2025)**

| **School** | **Baseline** | **Endline** |
| --- | --- | --- |
| **School A (small)** | ****Leafy greens****   - Amaranth (*Amaranthus spp*.) - Japanese malunggay (*Sauropus androgynus*) - Malabar spinach (*Basella alba*) - Moringa, lvs (*Moringa oleifera*) - Philippine spinach (*Talinum fruticosum*) - Water spinach (*Ipomoea aquatica*) - Wild Marjoram (*Origanum vulgare*)   **White roots, tubers, plantains**   - Cassava (*Manihot esculenta*) - Taro (*Colocasia esculenta*)   ****Other Vegetables****   - Blue ternate (*Clitoria ternatea* L.) - Bottle Gourd (*Lagenaria siceraria*) - Chili (*Capsicum annuum*) - Cucumber (*Cucumis sativus*) - Eggplant (*Solanum melongena*) - Onion chives (*Allium schoenoprasum*) - Roselle, lvs (*Hibiscus sabdariffa* L.) - Tomato (*Solanum lycopersicum*)   ****Vit A-rich Orange Vegetables****   - Sweet Potato (*Ipomoea batatas*)   ****Vit A-rich Fruits****   - Mango (*Mangifera indica*) - Papaya (*Carica papaya*)   **Other Fruits**   - Banana (*Musa* spp.) - Dragon fruit (*Hylocereus undatus*) - Jackfruit (*Artocarpus heterophyllus*) - Soursop (*Annona muricata*) - Strawberry (*Fragaria* × ananassa)   ****Pulses****   - Pigeon pea (Cajanus cajan) | ****Leafy greens****   - Amaranth (*Amaranthus spp*.) - Japanese malunggay (*Sauropus androgynus*) - Moringa, lvs (*Moringa oleifera*) - Philippine spinach (*Talinum fruticosum*) - Wild Marjoram (*Origanum vulgare*)   ****Other Vegetables****   - Blue ternate (*Clitoria ternatea* L.) - Bittergourd (Momordica charantia), - Bottle Gourd (*Lagenaria siceraria*) - Chayote (*Sechium edule*) - Chili (*Capsicum annuum*) - Cucumber (*Cucumis sativus*) - Eggplant (*Solanum melongena*) - Jute mallow (*Corchorus olitorius*), - Roselle, flower (*Hibiscus sabdariffa* L.) - Onion chives (*Allium schoenoprasum*)   ****Vit A-rich Fruits****   - Mango (*Mangifera indica*) - Papaya (*Carica papaya*)   **Other Fruits**   - Banana (*Musa* spp.) - Jackfruit (*Artocarpus heterophyllus*) - Soursop (*Annona muricata)*   **Pulses**   - Cowpea (Vigna unguiculata (L.) - **Rice Bean** (Vigna umbellate) - string beans,   **Nuts and Seeds**   - Sesame (*Sesamum indicum* L.) |
| **School B**  **(medium)** | **Leafy greens**   - **Amaranth (*Amaranthus spp.)*** - **Pechay (*Brassica rapa*)** - **Philippine spinach (*Talinum fruticosum*)**   **White roots, tubers, plantains**   - **Cassava (*Manihot esculenta*)**   **Other vegetables**   - **Chili, lvs and fruit (*Capsicum annuum*)** - **Eggplant (*Solanum melongena*)** - **Onion chives (*Allium schoenoprasum*)** - **Tomato (*Solanum lycopersicum*)** - **Wild Marjoram (*Origanum vulgare*)**   **Vitamin A rich fruits**   - **Mango (**Mangifera indica) - **Papaya, ripe (*Carica papaya*)**   **Other fruits**   - **Banana (*Musa* sp.)** - **Cotton fruit** (Sandoricum koetjape) - **Guava (*Psidium guajava*)**   **Pulses**   - **Pigeon pea (*Cajanus cajan*)** - **String beans (*Phaseolus vulgaris* L.)**   **Citrus**   - **Calamansi (*Citrus* × microcarpa)**   **Nuts and seeds**   - **Cacao (*Theobroma cacao*)** | **Leafy greens**   - **Japanese malunggay** (Sauropus androgynus) - **Malabar spinach** (Basella alba) - **Pechay** (Brassica rapa) - **Philippine spinach** (Talinum fruticosum) - **Water spinach** (Ipomoea aquatica)   ****Other vegetables****   - **Bitter gourd** (Momordica charantia) - **Eggplant** (Solanum melongena) - **Onion chives** (Allium schoenoprasum) - **String beans** (Phaseolus vulgaris) - **Squash** (Cucurbita spp.) - **Tomato** (Solanum lycopersicum) - **Wild Marjoram (**Origanum vulgare)   ****Vitamin A rich fruits****   - **Mango (**Mangifera indica) - **Papaya, ripe** (Carica papaya)   ****Other fruits****   - **Banana** (Musa sp.) - **Cotton fruit** (Sandoricum koetjape) - **Guava (*Psidium guajava*)** - **Mulberry** (Morus spp.)   **Nuts and seeds**   - **Sesame** (Sesamum indicum)   ****Pulses****   - **Cowpea** (Vigna unguiculata) - **Winged bean** (Psophocarpus tetragonolobus)   **Citrus**   - **Calamansi (*Citrus* × microcarpa)**   **Nuts and seeds**   - **Cacao (*Theobroma cacao*)** |
| **School C (large)** | **White roots, tubers, plantains**   - **Cassava (*Manihot esculenta*)**   **Leafy greens**   - **Amaranth (*Amaranthus* spp.)** - **Moringa, lvs (*Moringa oleifera*)** - **Water Spinach (*Ipomoea aquatica*)**   **Other vegetables**   - **Bottle gourd (*Lagenaria siceraria*)** - **Chili, lvs and fruit (*Capsicum annuum*)** - **Eggplant (*Solanum melongena*)** - **Tomato (*Solanum lycopersicum*)** - **Papaya, unripe (*Carica papaya*)**   **Vitamin A rich fruits**   - **Papaya, ripe (*Carica papaya*)**   **Other fruits**   - **Banana (*Musa* sp.)** - **Dragon fruit (*Hylocereus undatus*)** - **Mulberry (*Morus* spp.)** - **Soursop (*Annona muricata*)** | **Leafy greens**   - **Moringa, lvs** (*Moringa oleifera*)   **Other vegetables**   - Cucumber (*Cucumis sativus*) - **Eggplant (*Solanum melongena*)** - **Squash** (*Cucurbita maxima* L.) - **String bean** (*Phaseolus vulgaris*) - Papaya, unripe (***Carica papaya*)**   **Vitamin A rich fruits**   - **Papaya, ripe (*Carica papaya*)**   **Other fruits**   - **Banana (*Musa* sp.)** - **Mulberry (*Morus* spp.)** - **Soursop (*Annona muricata*)**   **Pulses**   - **Lima bean** (*Phaseolus lunatus* L.) |

***Note:*** *Both School A and School B maintained marigold plants from baseline to endline as a natural insect repellent.*
