# Supplementary material for: Fueling Bright Futures: a mixed-methods case study on integrating gardens, canteens, and nutrition training in philippine public schools
Source: BMC Public Health. 2026 Jul 29;26:3063. doi: 10.1186/s12889-026-28430-w (PMC13700392; doi:10.1186/s12889-026-28430-w)
Supplement: Supplementary file 5 [file 12889_2026_28430_MOESM5_ESM.docx]

# Supplementary Materials E

**Table S3: Select Examples of Menu Modifications Made to Improve Nutritional Quality of Hot Meals**

| School | Pre | Post | Improvements |
| --- | --- | --- | --- |
|  | **Original Recipe** | **Enhanced Recipe** |  |
| School A | Spaghetti: spaghetti noodles, canned corned beef, canned tomato sauce | Spaghetti: spaghetti noodles, rice beans (*Vigna umbellata*), canned tomato sauce | Replaced a highly processed fatty meat with a vegetable protein. |
| School B | *Pancit:* rice noodles, cabbage carrots, soy sauce, chicken, oyster sauce, and oil | *Pancit:* rice noodles, pehay, sitaw, chicken, and reduced amounts of soy sauce, oyster sauce, and oil. | Added nutrient-dense school garden produce; modified cooking practice to use less oil and salt. |
| School C | *Arroz Caldo*: glutinous rice and chicken stock | *Arroz Caldo:* glutinous rice, chicken stock, eggs, and malunggay (*Moringa oleifera*) | Added nutrient-dense school garden produce (*malunggay*), and protein-rich eggs. |
|  | *Champorado*: glutinous rice, cacao powder, sugar | *Champorado:* oatmeal, cacao powder, and reduced amounts of sugar | Replaced glutinous rice with nutrient-dense oatmeal and reduced the amount of sugar. |
